# An etiology‐driven framework for status epilepticus and ictal–interictal continuum

**DOI:** 10.1002/epi.70209

**Published:** 2026-03-18

**Authors:** Simona Lattanzi

**Affiliations:** ^1^ Neurological Clinic, Department of Experimental and Clinical Medicine Marche Polytechnic University Ancona Italy

To the Editors,

I congratulate Dr. Bosque‐Varela and colleagues for providing interesting insights into the relationships between peri‐ictal magnetic resonance imaging (MRI) abnormalities (PMA) and ictal–interictal continuum (IIC) patterns.^1^

In a prospective cohort study, 223 adult patients diagnosed with status epilepticus (SE) or IIC underwent electroencephalography (EEG) and MRI within 48 h of diagnosis. PMA occurrence aligned closely in patients with IIC (47%) and in patients with SE (43%). The latent cluster analysis revealed two distinct classes, primarily based on EEG patterns, their localization, and the etiology. Class 1 was associated with high‐frequency and nonunilateral periodic discharges (PD)/spike‐and‐waves or sharp‐and‐waves (SW) patterns and predominantly acute‐triggering factors in epilepsy (81%) as an etiology. In Class 2, low‐frequency unilateral PD/SW predominated along with a mixture of etiologies, mostly remote, progressive, or unknown (60%) followed by acute primary or secondary etiologies (34%). Interestingly, a statistically significant difference was found in the rates of PMA between the two classes, with PMA occurring more frequently in patients with low‐frequency PD/SW and symptomatic etiologies (Class 1: 18%, Class 2: 50%; *p* = 0.02).

The study did not simply challenge the strict separation of SE and IIC but also suggested considering etiology while interpreting EEG in patients with IIC. Acute symptomatic etiology encompasses a variety of heterogeneous causes, and four subcategories have been recently proposed, namely “acute‐triggering factors in epilepsy,” “acute primary central nervous system (CNS) pathology” (including cerebrovascular diseases, active CNS infections, or head trauma), “secondary CNS pathology” (including metabolic disturbances or systemic infection), and “drug or alcohol intoxication and withdrawal.”^2^ Differences in the risk of in‐hospital mortality, poor functional outcome at discharge, and post‐SE epilepsy have been identified among these subgroups.^2^, ^3^ Although the segregation of either PMA or EEG features according to the underlying etiological categories of SE/IIC reinforced the validity of the newly proposed classification system from a new perspective and through independent external data,^1^ it would have been interesting to adopt a more granular approach. Lumping acute primary and secondary CNS pathology and pooling together remote, progressive, or unknown etiologies may have prevented the opportunity to appreciate clusters with distinctive electroclinical phenotypes. Exploratory analyses based on different etiological groups might build further on the research question by identifying additional associations between variables and providing novel insights.

An etiology‐driven framework that considers distinct subcategories may better suit the heterogeneity and the continuum spectrum of severity and prognosis of suspected nonconvulsive SE, resulting in improved diagnostic accuracy and more individualized management strategies.^4^ Further studies are needed to develop a multidimensional systematization that integrates clinical, EEG, neuroimaging, and etiological axes.^5^, ^6^

## CONFLICT OF INTEREST STATEMENT

Simona Lattanzi has received speaker's or consultancy fees from Angelini Pharma, Eisai, GW Pharmaceuticals, Medscape, NewBridge Pharmaceuticals, and UCB Pharma and has served on advisory boards for Angelini Pharma, Arvelle Therapeutics, Bial, Eisai, GW Pharmaceuticals, Rapport Therapeutics, and UCB Pharma outside the submitted work. I confirm that I have read the Journal's position on issues involved in ethical publication and affirm that this report is consistent with those guidelines.

## Data Availability

Data sharing not applicable to this article as no datasets were generated or analysed during the current study.
